# TrisOxine abiotic siderophores for technetium complexation: radiolabeling and biodistribution studies

**DOI:** 10.1186/s41181-023-00214-2

**Published:** 2023-10-19

**Authors:** Julien Leenhardt, Alexandre Biguet Petit Jean, Florian Raes, Emilien N’Guessan, Marlène Debiossat, Clémence André, Sandrine Bacot, Mitra Ahmadi, Nicolas de Leiris, Loïc Djaileb, Catherine Ghezzi, Marie-Dominique Brunet, Alexis Broisat, Pascale Perret, Amaury du Moulinet d’Hardemare

**Affiliations:** 1 Université Grenoble Alpes, INSERM, CHU Grenoble Alpes, LRB, 38000 Grenoble, France; 2 CHU Saint-Etienne, Radiopharmacy/Nuclear Medicine, Saint-Etienne, France; 3 Université Grenoble Alpes, Department of Molecular Chemistry, Saint Martin d’Hères, France

**Keywords:** Technetium-99m, Radiochemistry, Siderophores, Chelates, Radiochemical purity, Oxidation state, Biodistribution, Nano SPECT/CT imaging

## Abstract

**Background:**

Despite the development of positron emission tomography (PET), single photon emission computed tomography (SPECT) still accounts for around 80% of all examinations performed in nuclear medicine departments. The search for new radiotracers or chelating agents for Technetium-99m is therefore still ongoing. *O-*TRENSOX and *O-*TRENOX two synthetic siderophores would be good candidates for this purpose as they are hexadentate ligands based on the very versatile and efficient 8-hydroxyquinoline chelating subunit. First, the radiolabeling of *O-*TRENOX and *O*-TRENSOX with ^99m^Tc was investigated. Different parameters such as the quantity of chelating agent, type of reducing agent, pH and temperature of the reaction mixture were adjusted in order to find the best radiolabeling conditions. Then an assessment of the partition coefficient by measuring the distribution of each radiosynthesized complex between octanol and phosphate-buffered saline was realized. The complex’s charge was evaluated on three different celluloses (neutral, negatively charged P81 and positively charged DE81), and finally in vivo studies with biodistribution and SPECT imaging of [^99m^Tc]Tc-*O-*TRENOX and [^99m^Tc]Tc-*O-*TRENSOX were performed.

**Results:**

The radiolabeling studies showed a rapid and efficient complexation of ^99m^Tc with both chelating agents. Using tin pyrophosphate as the reducing agent and a minimum of 100 nmol of ligand, we obtained the [^99m^Tc]Tc-*O-*TRENOX complex with a radiochemical purity of more than 98% and the [^99m^Tc]Tc-*O-*TRENSOX complex with one above 97% at room temperature within 5 min. [^99m^Tc]Tc-*O-*TRENOX complex was lipophilic and neutral, leading to a hepatobiliary elimination in mice. On the contrary, the [^99m^Tc]Tc-*O-*TRENSOX complex was found to be hydrophilic and negatively charged. This was confirmed by a predominantly renal elimination in mice.

**Conclusions:**

These encouraging results allow us to consider the *O-*TRENOX/^99m^Tc and *O-*TRENSOX/^99m^Tc complexes as serious candidates for SPECT imaging chelators. This study should be continued by conjugating these tris-oxine ligands to peptides or antibodies and comparing them with the other bifunctional agents used with Tc.

**Supplementary Information:**

The online version contains supplementary material available at 10.1186/s41181-023-00214-2.

## Background

Despite the development of PET imaging in the recent years, SPECT imaging still accounts for approximately 80% of all scans performed in nuclear medicine departments worldwide (Crișan et al. 2022; Israel et al. 2019). The ^99m^Tc radioisotope has optimal nuclear properties with a half-life of 6.02 h, permitting the synthesis, administration and collection of clinically relevant quality images. The 140 keV monochromatic gamma photon is ideal for high spatial resolution SPECT imaging. In addition, there is low radiation exposure to the patients and staff due to the absence of particle radiation. The on-site availability of a low-cost ^99^Mo/^99m^Tc generator system, which overcomes the problem of distribution worldwide, is an additional advantage (Brunello et al. 2022). Though lower resolution than PET imaging, the developments in SPECT imaging systems based on new solid-state cadmium telluride and zinc telluride (CZT) crystals and efficient collimator design led to an increase in resolution and sensitivity (Crișan et al. 2022). Finally, advances in the radiometals radiolabeling, particularly the development of radiopharmaceuticals for PET imaging (such as [^68^Ga]Ga-PSMA11), can be translated to radiometals used in SPECT imaging, such as technetium (Duatti 2021). The use of ^99m^Tc for SPECT imaging is therefore far from over.

Siderophores are endogenous or exogenous molecules able to bind iron selectively in its ionic form (Fe^3+^ ion) (Vaulont & Schalk 2015). Many siderophores are produced by the plants to capture iron from the soil. In both animals and humans, siderophores play more diverse roles: iron transport, iron capture to deprive bacteria of this element in case of infection, or even the removal of iron in excess responsible for toxicity on organs. Siderophores are also able to chelate various other metals such as Ca^2+^, Cu^2+^, Zn^2+^ and Al^3+^ ions, sometimes with less selectivity (Biaso et al. 2002). These molecules should therefore be able to complex a variety of other metal cations, including those used in nuclear medicine, such as Technetium-99m or Gallium-68. This is already the case for the latter with natural or artificial siderophores (Davey and Paterson 2022), and we can also mention Zirconium-89 with the new chelating agents derived from Desferrioxamine B (Salih et al. 2022).

**Fig. 1:**
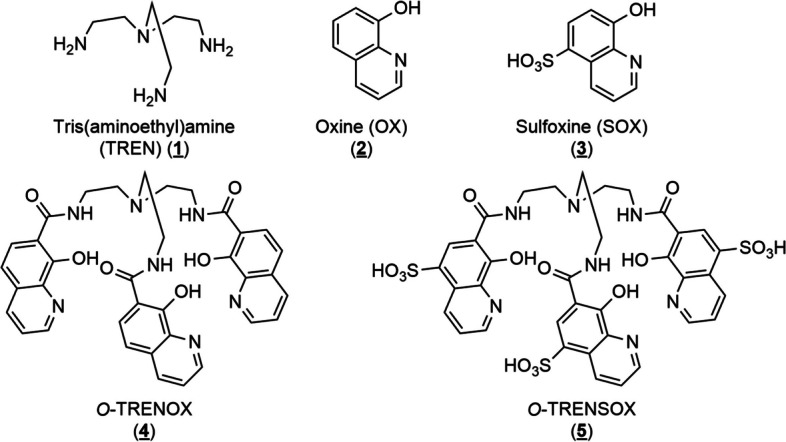
Chemical structure of tris(aminoethyl)amine (**1**), oxine (**2**), sulfoxine (**3**), *O-*TRENOX (**4**) and *O-*TRENSOX (**5**)

We synthesized two abiotic siderophores derived from tris(aminoethyl)amine (TREN (**1**); Fig. 1) and Oxine (OX (**2**); Fig. 1) or Sulfoxine (SOX (**3**); Fig. 1) namely *O-*TRENOX (**4**) (Fig. 1) and *O-*TRENSOX (**5**) (Fig. 1) with exceptional capability to bind very strongly Fe^3+^ (du Moulinet d’Hardemare et al. 2006). These two chemical structures only differ by the addition of a sulphonate group on the three oxines of *O-*TRENSOX compared to *O-*TRENOX. This provides two chelates with different lipophilic characteristics.

Complexation of Technetium-99m by bidentate oxine has already been described in the literature (Baldas and Bonnyman 1992; Colas-Linhart et al. 1983), as well as with Indium-111 and Gallium-67 (Hnatowich and Clancy 1980). Curiously, in the literature concerning radiopharmaceuticals, there are no examples of the use of tetra or hexadentate ligands based on oxine when they could ensure, for example, a stronger complexation and therefore a greater stability of the radiopharmaceuticals produced.

Herein, we report the radiolabeling with ^99m^Tc followed by characterization studies of these two hexadentate abiotic siderophores. We first investigated the optimal conditions for radiolabeling the siderophores *O-*TRENOX and *O-*TRENSOX with ^99m^Tc by varying numerous parameters (reducing agent, quantity of ligand, pH, and temperature). Then, we evaluated various characteristics of [^99m^Tc]Tc-*O-*TRENOX and [^99m^Tc]Tc-*O-*TRENSOX, including lipophilicity, charge determination*,* and in vivo biodistribution in mice.

## Methods

### Synthesis of *O-*TRENOX (4) and *O-*TRENSOX (5)

**Fig. 2:**
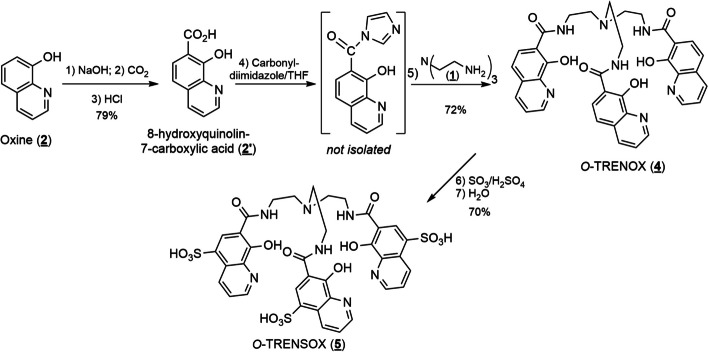
Synthesis of *O*-TRENOX (**4**) and O-TRENSOX (**5**)

Synthesis of the siderophores *O-*TRENOX (**4**) and *O-*TRENSOX (**5**) was previously carried out by our team (Baret et al. 1995; Caris et al. 1996; du Moulinet d’Hardemare et al. 2010). The synthesis has been reproduced with very few modifications following the Fig. 2.

Reagents are of commercial origin and used without further purification other than are indicated. Solvents are purified according to standard procedures. ^1^H NMR and ^13^C NMR were recorded using a Bruker Avance III 400. The chemical shift δ is given in ppm. High Resolution Mass Spectral data were obtained using an LTQ Orbital XL Thermo Scientific (ESI).

- 8-Hydroxyquinoline-7-carboxylic acid (**2’**)

In a flask, NaOH pellets (8 g; 0.2 mol) were dissolved in 250 ml methanol, then 8-hydroxyquinoline (**2**) (29 g; 0.2 mol) was added. After overnight stirring, the methanol was removed under reduced pressure and the resulting solid was carefully dried by heating at 60°C under reduced pressure for 2 h. The dried powder was rapidly transferred to a dry autoclave, then placed under CO_2_ pressure (50Bar) with heating at 160°C for 5 days. After cooling, the powder obtained is poured into 200 mL of water, the solution filtered and acidified to pH 2 with hydrochloric acid (HCl). The resulting precipitate was filtered and washed with cold water. The beige solid obtained in 79% yield was dried in a desiccator at 60°C (30 g; 0.15 mol).

^1^H NMR (400 MHz, DMSO-*d6*): *δ* = 7.33 (d, ^3^J = 7.96 Hz, 1H Ar), 7.82 (m, 1H, Ar), 7.94 (d, ^3^J = 7.96 Hz, 1H, Ar), 8.62 (d, 3J = 7.2 Hz, 1H, Ar), 8.94 (d, ^3^J = 2.9 Hz, 1H, Ar). ^13^C NMR (126 MHz, DMSO-*d6*): *δ* = 112.7, 115.1, 128.6, 133.1, 137.5, 140.7, 147.3, 161.7, 171.8 ppm. MS (ESI): m/z calcd for C10H7NO3: 189.04, found: 189.96 [M+H]^+^, 211.94 [M+Na]^+^, 171.95 [M-H_2_O]^+^.

- O-TRENOX (**4**)

Anhydrous THF (50 mL), 8-hydroxyquinoline-7-carboxylic acid (**2′**) (0.5 g; 2.64 mmol) and 1,1'-carbonyldiimidazole (0.47g; 2.91 mmol) were successively introduced into a dry flask under argon gas. After refluxing the solution for one hour, TREN (**1**) (tris(2-aminoethyl)amine, 0.13g; 0.88 mmol) was carefully added in solution in anhydrous THF (10 mL) using a syringe. After refluxing overnight under an argon atmosphere, the THF was evaporated under reduced pressure and the residue recovered in chloroform (100–150 mL). This solution was washed with a molar solution of ammonium chloride (2 × 30 ml), followed by distilled water (2 × 30 mL) and finally dried over anhydrous sodium sulfate. After evaporation of the chloroform, the solid was recrystallized in a water–methanol mixture to give *O*-TRENOX (**4**) (0.42g; 0.66 mmol) in 72% yield.

^1^H NMR (400 MHz, DMSO-*d6*): *δ* = 2.88 (pseudo t, 6H, N-CH_2_), 3.57 (m, 6H, CH_2_-NH-CO), 7.21 (d, ^3^J = 8.7 Hz, Ar), 7.58 (dd, ^3^J = 8.2 Hz,^4^J = 4.1 Hz, 1H, Ar), 7.91 (d, ^3^J = 8.7 Hz, 1H, Ar), 8.22 (d, ^3^J = 8.2 Hz, 1H, Ar), 8.82 (d, ^3^J = 4 Hz, 1H, Ar), 8.91 (s broad, -NH-CO). ^13^C NMR (126 MHz, DMSO-*d6*): *δ* = 38.4, 53.7, 113.7, 117.5, 124.2, 126.1, 131.2, 136.8, 139.8, 149.6, 157.0, 168.6 ppm. MS (ESI): m/z calcd for C36H33NO6N7: 659.24, found: 660.26 [M+H]^+^, 682.23 [M+Na]^+^.

- *O*-TRENSOX (**5**)

*O-TRENOX* (**4**) (0.5 g; 0.79 mmol) was added in portions to oleum (SO_3_/H_2_SO_4_; 15 mL) while stirring vigorously. After overnight stirring at room temperature, the mixture was carefully poured over ice to give a yellow precipitate which was quickly filtered and washed with a small amount of ice-cold water. The solid was recrystallized in a minimum amount of water, then filtered and dried at 30°C to give a yellow solid in 70% yield.

^1^H NMR (400 MHz, acidic form, D_2_O): *δ* = 3.75 (broad s, 6H, N-CH_2_); 3.97 (broad s, 6H, CH_2_-NH-CO); 7.79 (dd, ^3^J = 7.9 Hz, ^4^J = 5.2 Hz, 3H, Ar); 8.42 (s, 3H, Ar); 8.62 (d, ^4^J = 5.2 Hz, 3H, Ar); 8.90 (d, ^3^J = 7.9 Hz, 3H, Ar). ^I3^C NMR (126 MHz, sodium salt, D_2_O): *δ* = 39.9, 55.4, 126.3, 131.9, 141.5, 146.28, 115.54, 123.5, 129.0, 139.1, 164.80, 171.7 ppm. MS (ESI): m/z calcd for C36H33NO6N7S3: 659.24, found: 660.26 [M+H]^+^, 682.23 [M+Na]^+^. MS (ESI): m/z calcd for C36H33O6N7: 899.88, found: 900.89 [M+H]^+^.

### Radiolabeling of [^99m^Tc]Tc-*O-*TRENOX and [^99m^Tc]Tc-*O-*TRENSOX

*O-*TRENSOX (**5**) solution was prepared in ultra-pure water, while *O-*TRENOX (**4**) solution was produced with a mix of absolute ethanol (Carlo Erba reagents) and ultra-pure water (50:50; v/v). Concentrations obtained varied between 0.5 and 1 mg/mL (757.6 to 1515.2 nmol/mL). Sodium pertechnetate (T_1/2_ = 6.02 h, Eγ = 140 keV) was obtained from a freshly eluted ^99^Molybdenum / ^99m^Technetium generator (TEKCIS, Cis Bio). Amount of pertechnetate applied varied between 100 and 1,000 MBq in a volume of sodium chloride (NaCl) 0.9% ranging from 20 to 200 µL. Two reductive tin sources were used to reduce technetium to lower oxidation state: tin chloride and tin pyrophosphate. Ligand and reducer were introduced into a sterile vial. The pH was checked with paper (Machery-Nagel) and adjusted if necessary with a 0.05 M NaOH solution or a 0.5 M acetate buffer solution at pH 4. The solution was mixed and either heated or left at room temperature for several minutes.

Factors influencing radiosynthesis such as the amount of chelate (1–1000 µg to 1.52–1515.2 nmol), the amount of reducing agent (0.1–2000 µg) the presence of weak Technetium-99m complexing agents which would allow the stabilization of pertechnetate reduction intermediates (*e.g.* chloride, pyrophosphate or tartrate ions), the reaction time, pH of the solutions (4–10), and the reaction temperature (20–70 °C) were studied with *O-*TRENOX (**4**) and *O-*TRENSOX (**5**) in order to optimize the reaction conditions.

Moreover, the radiolabeling of *O-*TRENOX was also processed through [^99m^Tc]TcCl_6_^2−^ according to the following protocol: 0.5 mL of 12 M HCl and 0.1 mL of sodium pertechnetate (< 100 MBq) were added in a sealed vial. Temperature was set to 80°C for 30 min using a dry bath. After degassing and cooling, a thin layer chromatography (TLC) on Whatman® cellulose 1MM in 0.6 M HCl was done to ensure the formation of [^99m^Tc]TcCl_6_^2−^ in the mixture reaction (Rf (retention factor) = 1), whereas impurities remained at the deposition point. If the activity migration was greater than 90%, the hydrochloric acid was then completely removed by evaporation (20–30 min of heating at 90°C). The dry residue of [^99m^Tc]TcCl_6_^2−^ was redissolved in 200 µL of *O-*TRENOX (**4**) (300 nmol) in anhydrous acetonitrile, followed by 10 µL of an equimolar solution of triethylamine also in acetonitrile. This solution was evaporated to dryness and dissolved in 1 mL of chloroform. Finally, a double purification step was performed with the addition of 1 mL of distilled water to the reaction flask, mixing and separating the two immiscible phase layers manually.

### Quality controls

The radiochemical purity (RCP) of [^99m^Tc]Tc-*O-*TRENOX and [^99m^Tc]Tc-*O-*TRENSOX complexes were determined by radio-instant thin layer chromatography (radio-iTLC or radio-TLC) and radio-high-performance liquid chromatography (radio-HPLC).

TLC: The RCP of [^99m^Tc]Tc-*O-*TRENOX complex was assayed by radio-TLC on 8 × 1 cm strips of Whatman® 1MM pure cellulose paper. The eluent was a 4:1 chloroform (Fischer chemical): ethanol (VWR International) mixture. A drop (5 µL) of the complex was deposited 1 cm from the bottom of the support. After full migration along the support, the strips were counted with the mini-GITA radiochromatograph (BGO-V-detector). Free pertechnetate ([^99m^Tc]TcO_4_^−^) and reduced technetium ([^99m^Tc]TcO_2_) remained at the baseline of the strip while [^99m^Tc]Tc-*O-*TRENOX complex migrated to the solvent front of the TLC plate.

Radio-TLC was also performed to analyze [^99m^Tc]Tc-*O-*TRENSOX complex. Two complementary chromatographs were necessary in this case to isolate [^99m^Tc]TcO_4_^−^ and [^99m^Tc]TcO_2_: iTLC-SG (Agilent technologies) 10 × 1 cm strips were used for both cases. The first one used 0.9% NaCl as eluent: [^99m^Tc]TcO_2_ stayed at the baseline, while [^99m^Tc]TcO_4_^−^ and [^99m^Tc]Tc-*O-*TRENSOX moved up to the TLC plate. The second one used a methyl ethyl ketone (MEC VWR international)): ethyl-acetate (VWR international) 2:3 mixture; in this case, [^99m^Tc]TcO_2_ and [^99m^Tc]Tc-*O-*TRENSOX remained at the deposit point whereas [^99m^Tc]TcO_4_^−^ progressed to the top of the strip.

HPLC: RCP was confirmed by a Shimadzu HPLC chain comprising two LC-20AD pumps and a SPD-20A UV spectrophotometer set at 254 nm. This device was coupled to a LabLogic HPLC radio-detector. The column used was a Kinetex 5 µm XB-C18 100A, 50 × 2.1 mm. The radiometal complex used a modified HPLC gradient of A: ultra-pure water and B: CH_3_CN; 0–5 min: B = 10%, 5–8 min: 10% B to 100% B and 8–14 min: 100% B using a flow rate of 1 mL/min.

### Reaction kinetics and radiochemical stability in the reaction vial

The radiochemical yields of [^99m^Tc]Tc-*O-*TRENOX and [^99m^Tc]Tc-*O-*TRENSOX complexes were studied at different reaction times: 5, 30, 60, 180, 360 and 1440 min. This test provides two pieces of informations: the rate of complexation and its long-term radiochemical stability in the vial. For this purpose, after carrying out the radiolabeling, we evaluated the RCP at different times with radio-TLC and radio-HPLC.

### Determination of the partition coefficient (Log *P*)

The partition coefficient was determined by measuring the distribution of each radiosynthesized complex between octanol and phosphate-buffered saline (PBS), according to the protocol previously described by Virgone-Carlotta et al. (2016). Briefly, in a mixture of 500 µL octanol and 500 µL PBS, 20 µL of the complex were added to a vial. After a one-minute vortexing, the vial was centrifuged at 13,000 rpm for 3 min to ensure complete separation of the two phases. These two phases were separated manually, and then this process was repeated by supplementing the octanol phase with 500 µL of PBS and the aqueous phase with 500 µL of octanol. The phases were separated again and the 4 vials were counted separately using a gamma counter (COBRA, Packard). Log *P* was finally calculated as the ratio of the radioactivity in the octanol phase to the radioactivity in the PBS phase.

### Determination of the complex’s charge

In order to assess the complex's charge, TLC analyses were performed as described above, using 3 different celluloses: neutral Whatman® cellulose, ion exchange cellulose chromatography paper with negatively charged Whatman® P81 phosphate cellulose, and with positively charged Whatman® DE81 diethylaminoethyl-cellulose, and two mobile phases: chloroform: ethanol 80/20 mix for [^99m^Tc]Tc-*O-*TRENOX and methyl ethyl ketone: ethyl acetate 2:3 mix for [^99m^Tc]Tc-*O-*TRENSOX.

### In vivo studies: biodistribution and SPECT-CT imaging in mouse

All procedures were performed in accordance with the institutional guidelines and approved by the animal care and use committee of Grenoble University and the ad hoc French minister (APAFIS # 19480–2019022616164184 v4). Seven-week-old male Swiss mice were provided by Janvier Labs and housed in a temperature and humidity-controlled environment under a 12 h light–dark cycle with food and water ad libitum.

A 1:10 dilution in 0.9% NaCl was performed in order to reduce the amount of ethanol in the [^99m^Tc]Tc-*O-*TRENOX mixture to a maximum of 10% and to reach an appropriate volume activity (approximately 20–40 MBq in 100µL) for the mouse. For [^99m^Tc]Tc-*O-*TRENSOX, a 1:5 dilution in 0.9% NaCl was carried out to obtain a volume activity in a similar range to that of [^99m^Tc]Tc-*O-*TRENOX. The biodistribution study for each complex was performed in three groups (3 time points), each group contained four mice weighting 35 to 40 g. The radioactive solution was injected in one of the tail veins and the animals were euthanized by CO_2_ inhalation at 30, 60, and 240 min after injection. Blood samples were obtained by puncture of the heart, and urine samples by pressing the bladder. Other organs were harvested and samples were counted in a gamma counter (Wizard^2^, Perkin Elmer), and the result was expressed as a percentage of the injected dose (ID) per gram of organ (%ID/g).

Three mice in the 240 min group were also imaged at 30, 60, and 240 min before being euthanized to visualize the distribution of the complexes in vivo. SPECT/CT imaging was performed using a preclinical SPECT imaging camera (NanoSPECT/CT, Mediso) and the dedicated software Nucline (v3.04.010.0000, Mediso). Animals were placed on a pathogen-free animal handling system under gas anesthesia (isoflurane 1–2%). Animal temperature was maintained using a hot-air-channels and the respiratory frequency was continuously monitored using a sensor. Whole body SPECT imaging was performed for 25 min (32 s per projection). Mice were then put back in their cages to recover when necessary. Images were reconstructed, corrected for decay and expressed as a percent injected dose per cm^3^.

### Statistical analysis

Data were expressed as mean ± standard deviation (SD). Statistical analysis and graphical presentation were performed using GraphPad Prism version 6. Comparison between time points and between chelates were analyzed using a two-tailed nonparametric unpaired Man-Whitney test corrected for multiple comparison using Dunn’s test and using 2-ways ANOVA. Differences between groups were considered statistically significant if the P value was less than 0.05.

## Results

### Study of radiolabeling of [^99m^Tc]Tc-***O-***TRENOX and [^99m^Tc]Tc-***O-***TRENSOX

#### Type and quantity of reducer

The results obtained when stannous dichloride (SnCl_2_) was used as a reductant were not entirely satisfactory as the formation of a non-negligible (10% minimum) amount of hydrolyzed technetium [^99m^Tc]TcO_2_, identifiable on radio-TLC at Rf 0–0.2, was noticed. We therefore decided to use stannous pyrophosphate (Sn_2_(P_2_O_7_)) as reducing agent. As shown in Table 1, we obtained the best results with 25–50 µg of stannous pyrophosphate allowing to reach an excellent RCP, higher than 97% without the formation of [^99m^Tc]TcO_2_, along with less than 2% of [^99m^Tc]Tc-pyrophosphate complex*.* Conversely, with a very small amount of tin pyrophosphate, it was shown that the complexation did not occur.

**Table 1:** Variation of the radiochemical yield of [^99m^Tc]Tc-*O-*TRENOX and [^99m^Tc]Tc-*O-*TRENSOX determined by radio-HPLC as a function of the pyrophosphate amount

Pyrophosphate Sn (µg)	[^99m^Tc]Tc-*O-*TRENOX RCP (%)	[^99m^Tc]Tc-*O-*TRENSOX RCP (%)
0.1	4.0	2.6
0.5	50.0	6.5
1	93.9	28.8
5	96.6	73.9
25	98.5	97.6
50	98.7	98.2
100	97.9	96.6
500	96.5	95.6
1000	95.0	81.4
2000	78.0	75.1

Alternatively, for the complexation the intermediate [^99m^Tc]TcCl_6_^2−^ can be performed, on which chlorides are exchangeable ligands. With this method the formation of the [^99m^Tc]Tc-*O-*TRENOX complex was also obtained with the same analytical signature in HPLC and with a 96.7% RCP. However, unlike the previous labeling experiment using Sn-pyrophosphate, a purification step (extraction of the complex in chloroform) was required to increase the RCP to a very satisfactory level of 99%.

#### Amount of ligand

**Fig. 3:**
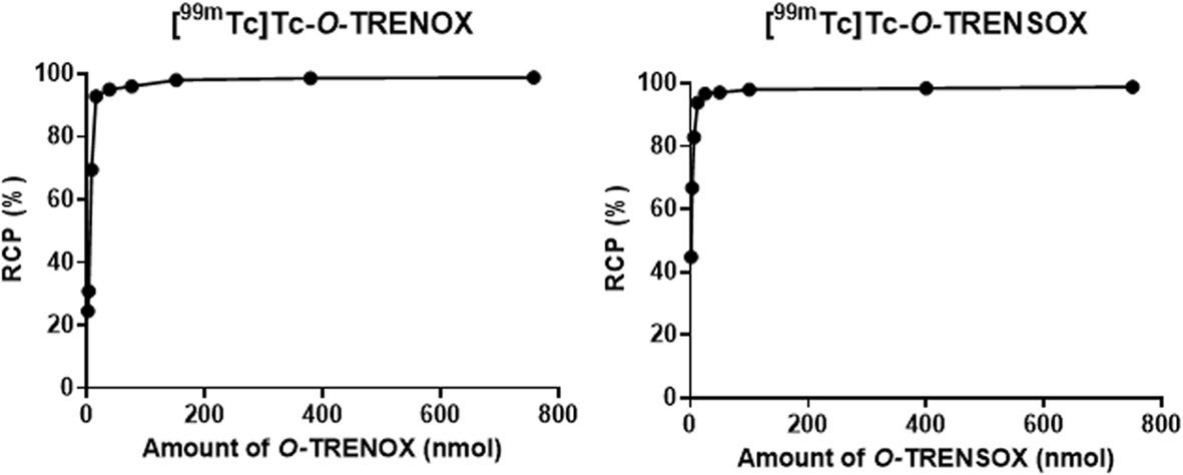
Variation of the radiochemical purity of [^99m^Tc]Tc-*O-*TRENOX and [^99m^Tc]Tc-*O-*TRENSOX as a function of *O-*TRENOX (**4**) and *O-*TRENSOX (**5**) amount (nmol)

The objective of this test was to vary the amount of *O-*TRENOX (**4**) and *O-*TRENSOX (**5**) in order to determine the minimal amount required and sufficient to obtain a high reaction yield. The results are shown in Fig. 3, the radiochemical purity was found satisfactory (RCP > 95%) with at least 35.9 nmol (25 µg) of *O-*TRENOX (**4**), and for a quantity of ligand equal to 151.5 nmol (100 µg) a maximum radiochemical purity plateau of 98% was obtained. The evaluation of the RCP as a function of the amount of *O-*TRENSOX (**5**) was also performed and the results were similar. From 25 nmol of *O-*TRENSOX (**5**) the RCP obtained was greater than 95% and for a quantity of ligand equal to 101 nmol (100 µg) a maximum radiochemical purity plateau of 98% was obtained.

#### Effect of pH

RCP data of [^99m^Tc]Tc-*O-*TRENOX and [^99m^Tc]Tc-*O-*TRENSOX obtained at different pH values are reported in Table 2. Maximum radiochemicals yield was obtained at pH 6, which was the pH of the reaction without adjustment. When the radiolabeling was performed at pH 4, the radiochemical yield dropped to 93.7% for [^99m^Tc]Tc-*O-*TRENOX and 92.4% for [^99m^Tc]Tc-*O-*TRENSOX.We also noticed a strong decrease in the radiochemical purity at pH higher than 6 for both chelates.

**Table 2:** Effect of reaction medium’s pH on the radiochemical yield of [^99m^Tc]Tc-*O-*TRENOX measured by iTLC

pH value	[^99m^Tc]Tc-*O*-TRENOX RCP (%)	[^99m^Tc]Tc-*O*-TRENSOX RCP (%)
4	93.7 ± 2.5	92.4 ± 1.5
6	98.3 ± 0.6	97.5 ± 0.7
8	78.2 ± 5.9	73.4 ± 2.5
9	67.3 ± 2.5	53.7 ± 3.2
10	29.5 ± 0.7	50.1 ± 5.7

Data are expressed in percentage as mean ± SD (*n* = 3)

#### Temperature

The effect of the temperature on the complexation efficiency was evaluated for the [^99m^Tc]Tc-*O-*TRENOX and [^99m^Tc]Tc-*O-*TRENSOX complex. For each test, one radiolabeling was performed at room temperature and compared to another one performed at 75°C for 20 min. Heating was found to not improve the radiochemical yield of both complexes. For the following experiments, the reaction temperature was then defined as room temperature (RT).

The TLC or HPLC radiochromatograms profiles of the [^99m^Tc]Tc-*O-*TRENOX and [^99m^Tc]Tc-*O-*TRENSOX complex were presented in the Additional file 1: Figs. S1, S2 and S3.

### Reaction kinetics and radiochemical stability in the reaction vial

The results of this test (Table 3) highlighted a rapid complexation of *O-*TRENOX with [^99m^Tc]TcO_4_^−^ since the radiochemical purity of the complex was 98.3 ± 0.2% after only 5 min of reaction. Moreover, the complex was stable for at least 24 h as the RCP remained elevated at 24h post-reaction (96.0 ± 0.5%). The results obtained for *O-*TRENSOX (**5**) were similar to those obtained with *O-*TRENOX (**4**), with an excellent radiochemical purity from 5 min of reaction at room temperature up to 24h post-radiolabeling in the reaction mixture.

**Table 3:** Effect of the reaction time on the radiochemical yield of [^99m^Tc]Tc-*O-*TRENOX and [^99m^Tc]Tc-*O-*TRENSOX measured by iTLC

Reaction time (min)	[^99m^Tc]Tc-*O-*TRENOX RCP (%)	[^99m^Tc]Tc-*O-*TRENSOX RCP (%)
5	98.3 ± 0.2	97.6 ± 0.8
30	98.5 ± 0.2	95.9 ± 0.1
60	98.0 ± 0.4	95.9 ± 0.6
180	97.4 ± 0.4	95.8 ± 1.0
360	96.6 ± 1.4	96.3 ± 0.8
1440	96.0 ± 0.5	95.2 ± 0.4

Data are expressed in percentage as mean ± SD (*n* = 3)

### Complex’s partition coefficient

The calculated log *P* for the [^99m^Tc]Tc-*O-*TRENOX complex was 1.2 ± 0.2, indicating a lipophilic behavior. In contrast, log *P* of the [^99m^Tc]Tc-*O-*TRENSOX complex was -2.3 ± 0.2.

### Complex’s charge

The results of the ion exchange chromatography are reported in Table 4. [^99m^Tc]Tc-*O-*TRENOX complex migrated equally on the three different supports. The complex was therefore probably neutral. For *O-*TRENSOX (**5**), the radiolabeled complex was retained by the positive cellulose DE81 while it migrated to the neutral cellulose as well as the negative cellulose P81. We concluded that the complex was negatively charged.

**Table 4:** Complex migration on different TLC supports

	P81 negative		DE81 positive		Whatman®neutral	
	Rf = 0 (%)	Rf = 0.8–1 (%)	Rf = 0 (%)	Rf = 0.8–1 (%)	Rf = 0 (%)	Rf = 0.8–1 (%)
[^99m^Tc]Tc-*O-*TRENOX	10.5 ± 2.3	89.5 ± 2.3	9.5 ± 2.5	90.5 ± 2.5	2 ± 0	97.3 ± 0.6
[^99m^Tc]Tc-*O-*TRENSOX	8.5 ± 1.7	91.8 ± 2.0	95.3 ± 5.5	4.8 ± 5.5	11 ± 2.8	89.0 ± 2.8

Data are expressed in percentage as mean ± SD (*n* = 3)

### In vivo studies: biodistribution and SPECT-CT imaging

**Fig. 4:**
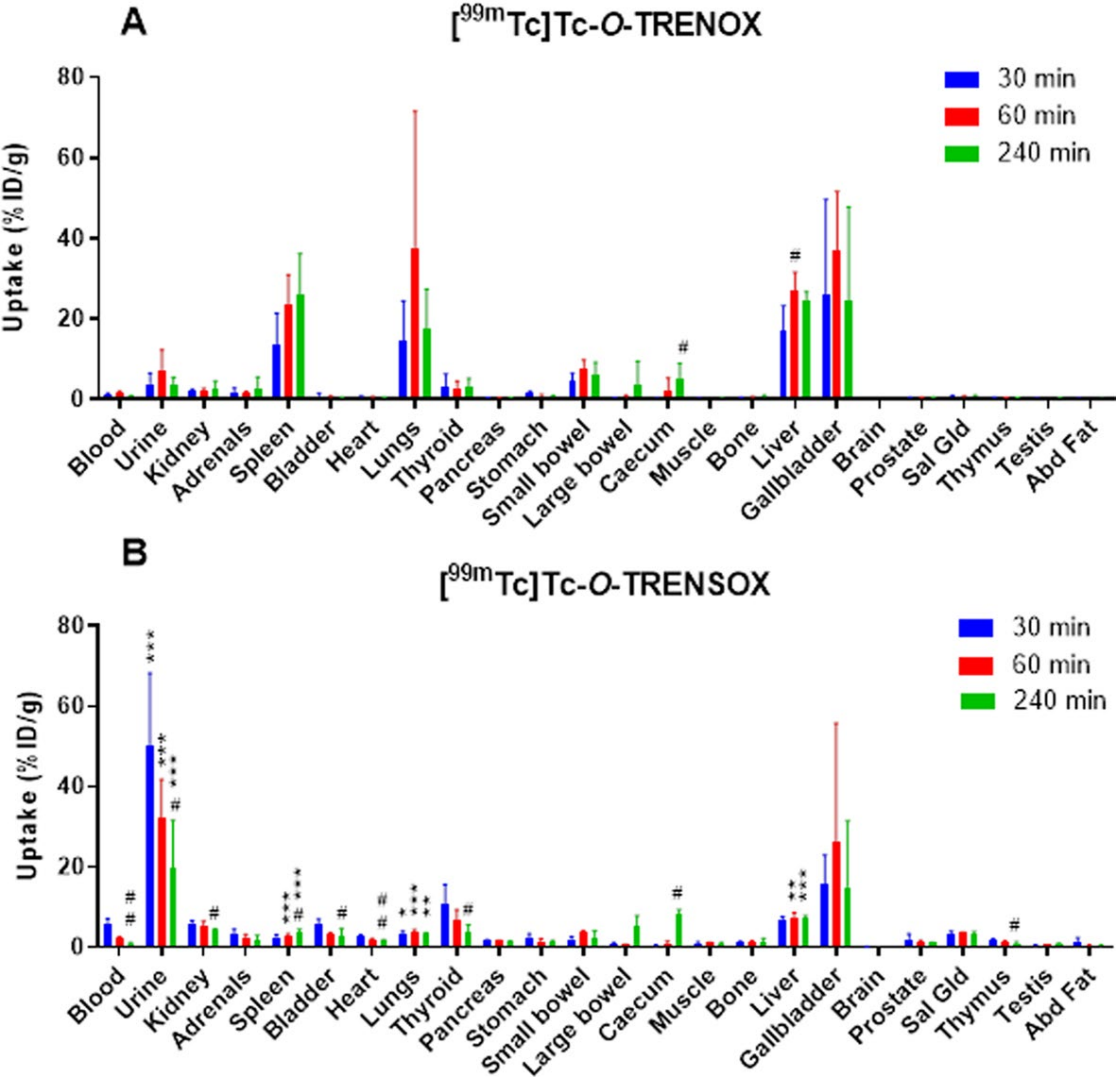
Biodistribution of [^99m^Tc]Tc-*O-*TRENOX (A) and [^99m^Tc]Tc-*O-*TRENSOX (B) in Swiss mice at 30, 60 and 240 min post-injection. Data are expressed in %ID/g as mean ± SD (*n* = 4 / time point). Abd: abdominal; Sal Gld: salivary glands. *: *p* < 0.05; **: *p* < 0.01 and ***: *p* < 0.001 versus [^99m^Tc]Tc-O-TRENOX. #: *p* < 0.05 and ##: *p* < 0.01 versus 30 min

[^99m^Tc]Tc-*O-*TRENOX and [^99m^Tc]Tc-*O-*TRENSOX biodistribution are presented in Figs. 4A and 3B, respectively. A side by side comparison of these chelators biodistributions at 30, 60 and 240 min is also available in Additional file 1: Figure S4. [^99m^Tc]Tc-*O-*TRENSOX injected dose was of 24.1 ± 11.1 MBq, in comparison with 11.1 ± 4.0 MBq for [^99m^Tc]Tc-*O-*TRENOX. This difference was attributable to the lipophilic nature of [^99m^Tc]Tc-*O-*TRENOX that resulted in a higher activity retained on empty syringes. In accordance with this lipophilic behavior, [^99m^Tc]Tc-*O-*TRENOX complex exhibited a predominantly hepatic distribution that was significantly higher than that of [^99m^Tc]Tc-*O-*TRENSOX at 60 min (27.08 ± 4.58 vs 7.35 ± 1.26%ID/g, *p* < 0.01) and at 240 min (24.61 ± 2.09 vs 7.22 ± 0.72%ID/g, *p* < 0.001) (Fig. 4). Conversely, [^99m^Tc]Tc-*O-*TRENSOX, which is more hydrophilic, was mainly eliminated by the kidneys as demonstrated by the fact that the highest activity was observed in the urine at all investigated time points (*p* < 0.001 vs [^99m^Tc]Tc-*O-*TRENOX). However, [^99m^Tc]Tc-*O-*TRENSOX was also, in a lesser extent, eliminated through the hepatobiliary system, as demonstrated by the elevated uptake in the gallbladder (max 15.06 ± 16.4%ID/g at 60 min) and the significant accumulation in the caecum (*p* < 0.05 between 30 and 240 min time points). A significance difference between the two chelates was also observed in the spleen. In this organ, the uptake of [^99m^Tc]Tc-*O-*TRENOX was of 23.62 ± 7.34 and 26.00 ± 10.26%ID/g at 60 and 240 min, respectively (*p* < 0.001 vs [^99m^Tc]Tc-*O-*TRENSOX). Finally, a significantly higher uptake of [^99m^Tc]Tc-*O-*TRENOX was also observed in the lungs at all time points. Interestingly, the longer the [^99m^Tc]Tc-*O-*TRENOX remained in vial after being diluted for the purpose of intravenous injections, the greater the pulmonary uptake was (Additional file 1: Fig. S5). Therefore, the lung uptake was attributed to a loss of solubility of this chelator following its dilution, leading to the formation of aggregates. Both chelators exhibited a rapid blood clearance with 0.85 ± 0.17 and 1.07 ± 0.19%ID/g remaining at 240 min (*p* = NS between chelators). Thyroid and stomach uptakes were minimal, suggesting an absence of free ^99m^Tc, and therefore a good in vivo stability of the two complexes. The lowest uptake was observed in the brain for both chelators and at all investigated time points.

**Fig. 5:**
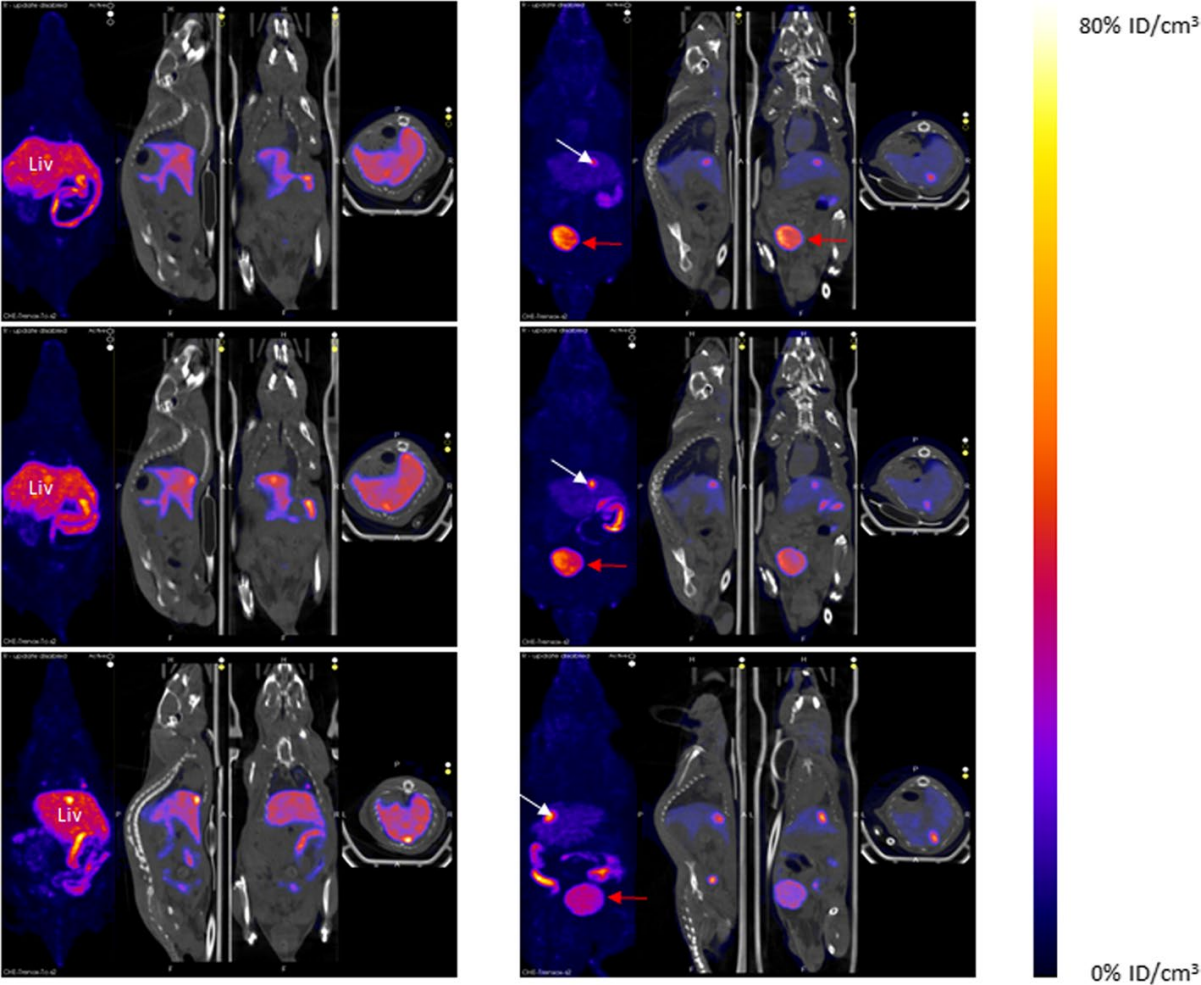
Representative SPECT/CT whole body imaging in Swiss mice 30, 60 and 240 min after [^99m^Tc]Tc-*O-*TRENOX (left) and [^99m^Tc]Tc-*O-*TRENSOX (right) injection. Images are expressed in % ID/cm^3^ as for biodistribution, to ensure that the values obtained are comparable. The three straight images represent the different sections: sagittal, coronal and transversal with a SPECT/CT reconstruction, while the image on the fare left represents the 3D reconstruction of the SPECT only. Liv: Liver, white arrow: Gallbladder, red arrow: Urine

On images in Fig. 5, on the left panel, [^99m^Tc]Tc-*O-*TRENOX at the 30-min time point was observed mainly in the liver, intestine and gallbladder of the mouse. The latter was clearly visible and the contrast intensified with time, in front maximum at 240 min. The visible contents in the intestine shifted over time. According to values obtained per organ after their collection, the spleen was also highly charged with 26.00 ± 10.26%ID/g at 4 h. However, the spleen was not easily distinguishable from the liver and intestines on SPECT image.

On the right panel of the Fig. 5 is imaging of the ^99m^Tc-*O-*TRENSOX complex in mice at different times. The bladder was clearly recognizable and was present in all imaging times. Gallbladder was also visible and increasingly active over time. Otherwise, there was limited hepatic and intestinal uptake, including the caecum.

The results obtained in this study are summarized in Table 5.

**Table 5:** Summary of the results obtained for the two complexes: [^99m^Tc]Tc-*O-*TRENOX and [^99m^Tc]Tc-*O-*TRENSOX

Chelate	Duration of synthesis at RT (min)	Solvent use	Mean RCP (%)	Mean RCP at 24 h (%)	Charge	Log *P*	Elimination
*O-*TRENOX (**4**)	5	Ethanol	98.3 ± 0.2	96.0 ± 0.5	Neutral	1.2 ± 0.2 Lipophilic	Hepatic and gallbladder
*O-*TRENSOX (**5**)	5	Water	97.6 ± 0.8	95.2 ± 0.4	Negative	− 2.3 ± 0.2 Hydrophilic	Urinary and gallbladder

## Discussion

The development of new bifunctional chelates for nuclear medicine and more particularly for theranostic applications (^99m^Tc/^188^Re—^68^Ga/^177^Lu) is still of great interest and topical. This manuscript describes radiolabeling studies of two abiotic siderophores (*O-*TRENOX (**4**) and *O-*TRENSOX (**5**)) with technetium-99m. The first part of this work consisted in finding the optimal conditions for radiolabeling of the *O-*TRENOX and *O-*TRENSOX chelate with technetium-99m. First, the best reducer had to be selected. The stannous ion Sn^2+^ is the reductant of choice for the formation of technetium complexes. Indeed, it reduces TcO_4_^−^ over a wide range of oxidation states (mainly at Tc + V but can also reach Tc + I) and Sn^2+^ is also validated for the constitution of radiopharmaceutical kits (Motaleb et al. 2011, 2018). Stannous dichloride was therefore initially used for the first radiolabeling with technetium-99m. However, the formation of a small amount of [^99m^Tc]TcO_2_ (“hydrolyzed technetium”) was observed. This specie is no longer available for complexation which limits the radiochemical purity here to 90%. In this context, the use of stannous pyrophosphate (Sn_2_(P_2_O_7_)) instead of stannous dichloride can avoid the formation of [^99m^Tc]TcO_2_ and may improve the radiochemical purity as shown by the work of Apparu et al. (Apparu et al. 1992). Indeed, the stannous pyrophosphate reacts with the [^99m^Tc]TcO_4_^−^ ion to form a [^99m^Tc]Tc-Pyrophosphate complex, on which a transchelation with the chosen ligand takes place. This exchange method is preferable when direct complexation reactions (such as with SnCl_2_) are relatively slow and thus avoids the formation of [^99m^Tc]TcO_2_. The use of HCl is another way to reduce the pertechnetate ion, although it is not commonly used in routine clinical practice. [^99m^Tc](TcCl_6_)^2−^ is formed on which an exchange with the chosen ligand can be attempted as mentioned in the equation below:

$$\begin{aligned} \left[ {^{{{\text{99m}}}} {\text{Tc}}} \right]{\text{TcO}}_{4}^{ - } + {\text{ HCl}}_{{{\text{excess}}}} & \to \, \left[ {^{{{\text{99m}}}} {\text{Tc}}} \right]\left( {{\text{TcCl}}_{{6}} } \right)^{{{2} - }} + O - {\text{TRENOX}} \\ & \to \left[ {^{{{\text{99m}}}} {\text{Tc}}} \right]{\text{Tc}}\left. {\left( {O - {\text{TRENOX}}} \right)} \right] + {\text{6 Cl}}^{ - } \\ \end{aligned}$$

After the neutralization of HCl in excess, the only other chemical species present in the reduced technetium solution was NaCl, which was contained in the eluate obtained from the generator (Na[^99m^Tc]TcO_4_^−^). There were therefore no stannous ions in the medium avoiding the formation of a low affinity intermediate complex as [^99m^Tc]Tc-metallic cation radio-colloid (Sundrehagen 1982). In addition, this method would provide only one species of reduced technetium, and therefore only one oxidation state (Liverant and Wolf 1982; March et al. 2005). The amount of ligand is also essential to ensure proper complexation with ^99m^Tc and depends on the chelate structure. Amounts of 6.2–30.8 nmol were required for [^99m^Tc]Tc-Sn (II) complexes of a novel quinoline derivative (Sanad et al. 2017) and 5.2 µmol for another ^99m^Tc quinoline complex (Motaleb et al. 2011). In the case of an inadequate ligand amount, the reduction of pertechnetate in an aqueous solution leads to cationic species evolving rapidly towards the formation of [^99m^Tc]TcO_2_ or its hydroxylated forms. Another important parameter is the pH of the reaction medium. As described by Motaleb et al. (Motaleb et al. 2011) and others (Akbar 2018; El-Kawy and García-Horsman 2017; Sanad et al. 2017), pH influences the formation of chelate-technetium species. This could be explained by the fact that an alkaline environment tends to favor the formation of stannous hydroxide colloids (El-Kawy & García-Horsman 2017). The finding of a lipophilic ([^99m^Tc]Tc-*O-*TRENOX) and a hydrophilic ([^99m^Tc]Tc-*O-*TRENSOX) complex is an important element. Indeed, this could be an advantage later on when the chelate will be linked to a molecule of interest in order to modify the lipophilic character of the final radiotracer. This difference in lipophilicity can be explained by the presence of sulfonic acids (–SO_3_H) groups grafted onto the quinoline ring of *O*-TRENSOX (**5**), which ionize completely in water to the sulfonate groups (–SO_3_^−^) and improve aqueous solubility (Baldas and Bonnyman 1992).

**Fig. 6:**
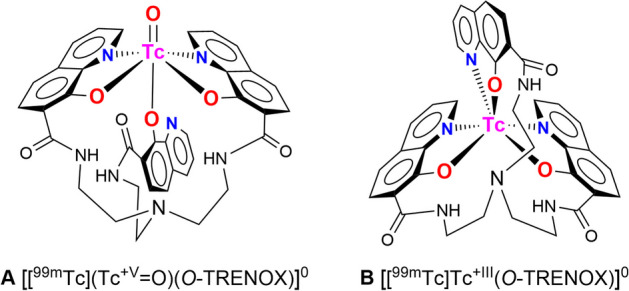
Proposed structures for the complex [^99m^Tc]Tc-*O*-TRENOX: **A** in the Tc+V oxidation state {[^99^Tc](Tc=O)^3+^} core and **B** in the Tc+III oxidation state

The overall charge of the complex formed between the technetium and the chelate could also provide information on the oxidation state of the metal center and on the nature and number of ligand atoms involved in the coordination. The charge of the complexes results from the balance of the charges brought by the ligand (and thus its protonation state) and those brought by the metal center. As the *O-*TRENOX (**4**) ligand does not contain sulfonate groups, only the phenol functions modulate the charge by deprotonation upon the complexation (up to 3 anionic charges; the tripodal nitrogen is unprotonated at pH 7). On the other hand, there are very few structural data on complexes between the technetium and the simple oxines, and no example of complexation with *bis*- or *tris*-bidentate oxines. In the first case, the representative work published by Wilcox et al. demonstrates the formation of [^99^Tc][Tc^V^O(Ox)_2_Cl] species, in which the metal is in the +V oxidation state with a {[^99^Tc]Tc=O^3+^} core and a chloride ion in the “*trans*” position (Wilcox et al. 1984). This non-redox reaction is achieved by the simple ligand exchange from [^99^Tc][Tc^+V^OCl_4_]^−^ and a bidentate oxine. If the same applies to *O*-TRENOX (**4**), as the complex is neutral, the proposed structure [^99m^Tc][Tc^+V^O(TRENOX)]° is shown in Fig. 6A, where three phenolates exactly compensate for the charge of the {[^99m^Tc]Tc=O^3+^} oxotechnetate core with but in which the ligand is only pentadentate (one of the three quinoline nitrogen does not participate in the complexation). A second structure may also be considered. In fact, the conditions employed by Wilcox et al. (1984) differ substantially from ours since we do not form intermediates in the +V oxidation state ([^99m^Tc] [Tc^V^OCl_4_]^−^, which moreover cannot be formed at the tracer level), and we are in reducing conditions (stannous ions in excess). In this case, we can envisage the structure [^99m^Tc][Tc^+III^(*O*-TRENOX)]° shown in Fig. 6B, in which the technetium is in the (+III) oxidation state. Indeed, the stannous ion is capable of forming Tc(+III) as in [^99m^Tc]Tc-EDTA complex (Seifert et al. 2004). In this proposed structure the ligand as above is *tris*-phenolate and compensates for the technetium charge in agreement with a neutral specie. One of the strengths of this structural hypothesis is that the ligand fully exerts its *tris*-bidentate ligand capacity as expected.

**Fig. 7:**
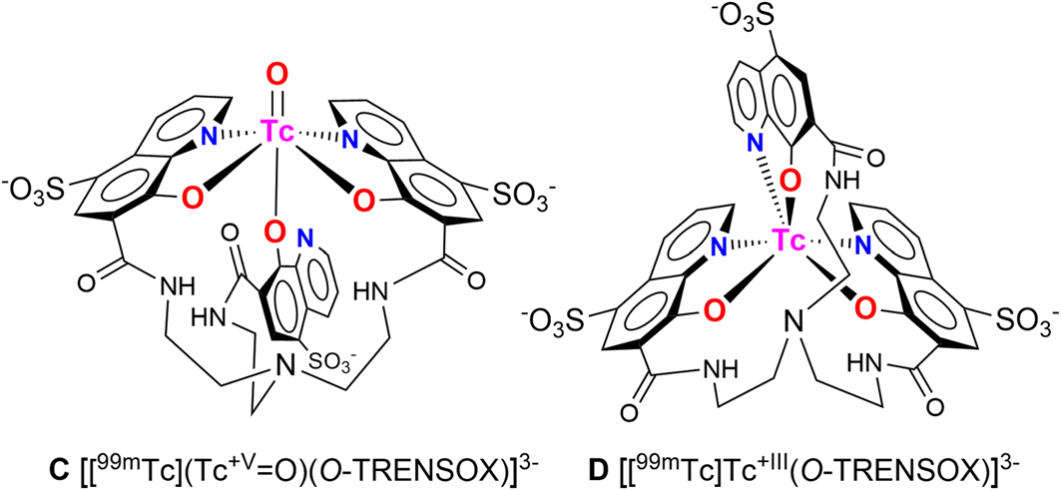
Proposed structures for the complex [^99m^Tc]Tc-*O*-TRENSOX: **C** in the Tc+V oxidation state {[^99^Tc](Tc=O)^3+^} core and **D** in the Tc+III oxidation state

Sulfonate groups are known to play a negligible role in coordination chemistry in water (Davies et al. 1996). Therefore, the hypothetical structures **C** (with a Tc=O^3+^ core in the +V oxidation state) and **D** (with Tc in the +III oxidation state) of the complex formed with *O*-TRENSOX and ^99m^Tc, must be identical to those of *O*-TRENOX and are shown in Fig. 7.

The only difference from the [^99m^Tc]Tc-*O*-TRENOX complex is that the complex formed this time has an anionic charge due to the total ionization of the sulfonic acids into 3 sulfonate groups. This result is consistent with the retention of this complex on DE81 anion exchange chromatography paper. In the absence of structural data using the long-lived and beta-emitting isotope ^99^Tc, it is not possible to discriminate between the hypothesis that the Tc is in the +V oxidation state (with the core Tc=O) and the hypothesis that Tc is in another oxidation state.

It should be noted that the experiment to form the [^99m^Tc]Tc-*O*-TRENOX complex from [^99m^Tc](Tc^+IV^Cl_6_)^2−^ which is in the Tc(+IV) oxidation state, does not allow us to decide more on the two structural hypotheses. Indeed, it is known that Tc(+IV)-Oxine complexes are not stable (Hwang et al. 1984) and that unstable Tc(+IV) complexes rapidly evolve by dismutation towards the +V and +III degrees, and these complexes are here indistinguishable.

Turning now to the biodistribution perspective, the global charge of a complex is a factor influencing the excretion or in *vivo* behavior in a significant way (Owunwanne et al. 1977): charged complexes tend to be eliminated by the kidneys, unlike the neutral complexes. This was exactly the case in the biodistribution studies in mice. The negatively charged [^99m^Tc]Tc-*O-*TRENSOX complex was eliminated mainly by the renal pathway and was present in the urine as early as 30 min after the injection. Conversely, the neutral [^99m^Tc]Tc-*O-*TRENOX complex had the characteristics to be an excellent radiotracer specific to the hepatobiliary system since it showed the following features: a rapid extraction from the plasma, a high biliary uptake, a little or no absorption from the intestine and a minimal concentration in the urinary tract (Akbar 2018; Wistow et al. 1977). The reason behind the lack of biodistribution in the brain despite the lipophilic nature of the [^99m^Tc]Tc-*O-*TRENOX complex could be due to an excessively high molecular weight of the complex (Waterhouse 2003) or to the absence of functional groups which ionize strongly at physiological pH, preventing the crossing of the blood–brain barrier. Concerning the presence of [^99m^Tc]Tc-*O-*TRENOX in the lungs, our hypothesis to explain this phenomenon is that the dilution made to be compatible with the iv injection to the mice (10% EtOH) led us to reduce the percentage of ethanol below the optimal concentration, and that therefore aggregate occurred formation over time. These small aggregates after injection in mice were then trapped in the capillaries of the microcirculation due to their size. The injection being performed intravenously, the first capillary bed to receive the injected complex and potential aggregates is that of the lungs. In support of this hypothesis, the supplementary material file Additional file 1: Figure S5 shows representative SPECT/CT imaging with small focal uptakes in the lungs. Moreover, this supplementary figure also shows the high correlation between the time post-dilution prior to IV injection and the lung uptake. The lack of biodistribution in the thyroid and stomach expressing the Na/I symporter, that is well known to transport free ^99m^Tc[TcO_4_^−^], confirmed the absence of a major decomplexation or demetallation of both complexes in mice, even after 4 h (Zhang et al. 2018). Moreover, the blood clearance was very rapid for both chelates with very low activity found in the blood. In conclusion, the biodistribution results in mice were promising for the stability of the complexes in vivo and the future development of these chelates.

## Conclusions

In summary, we have developed a fast and efficient radiolabeling of two chelates *O*-TRENOX and *O*-TRENSOX with technetium-99m. The technetiated complexes are obtained at room temperature with an excellent RCP and are stable both in vitro and in vivo. This makes it possible to consider their use as SPECT agents by deriving them into bifunctional agents. Depending on the nature of the radiotracer in development, we could select either *O*-TRENOX or *O*-TRENSOX: tracers for the brain imaging or myocardial perfusion for example, require a minimum degree of lipophilicity. On the other hand, tracers developed for oncology need 100% renal elimination, which implies the use of a hydrophilic tracer. These results also constitute the first example of the effective use of *tris*-bidentate oxine ligands in the complexation of technetium-99m, leading to much more stable complexes than simply bidentate oxines. This study should be continued by conjugating these tris-oxine ligands to peptides or antibodies and comparing them with the other bifunctional agents used with Tc, with a view to also studying radiolabeling with ^188^Re as part of theranostic pair formation. 

### Supplementary Information

**Additional file 1. Fig. S1**. [^99m^Tc]Tc(*O-*TRENOX)] chromatographic analysis on Whatman 0.16MM paper in a 4:1 Chloroform – Ethanol mixture. **Fig. S2**. HPLC radiochromatogramm of [^99m^Tc]Tc(*O-*TRENOX)] (radioactive signal on the top and UV signal on the bottom). **Fig. S3**. ([^99m^Tc]Tc(*O-*TRENSOX)] chromatographic analysis on iTLC SG paper with sodium chloride 0.9% (top) and in a 3:2 ethyl-acetate – Methyl ethyl ketone (bottom). **Fig. S4**. Comparative biodistribution of [^99m^Tc]Tc-*O-*TRENOX and [^99m^Tc]Tc-*O-*TRENSOX at each times (30, 60 and 240 minutes) (4 mice per chelate per times) formulated in %ID/g. *: p<0.05; ** p<0.01 and *** p<0.001 [^99m^Tc]Tc-*O-*TRENOX vs and [^99m^Tc]Tc-*O-*TRENSOX. **Fig. S5**. Representative SPECT-CT whole body imaging (sagittal, coronal and transversal views) with [^99m^Tc]Tc-*O-*TRENOX at 240 min after injection (A) and [^99m^Tc]Tc-*O-*TRENOX pulmonary uptake function of time from radiolabeling (B). White arrow: small spot in lungs, yellow arrow: liver, green arrow: intestine.

## Data Availability

All data generated or analyzed during this study are included in this published article.

## Abbreviations

PET: Position emission tomography
SPECT: Single photon emission computed tomography
TLC: Thin layer chromatography
Rf: Retention factor
RCP: Radiochemical purity
Radio-iTLC: Radio-instant thin layer chromatography
Radio-HPLC: Radio-high performance liquid chromatography
PBS: Phosphate-buffered saline
ID: Injected dose
