# Supplementary material for: TrisOxine abiotic siderophores for technetium complexation: radiolabeling and biodistribution studies
Source: EJNMMI Radiopharm Chem. 2023 Oct 19;8:32. doi: 10.1186/s41181-023-00214-2 (PMC10587049; doi:10.1186/s41181-023-00214-2)

TrisOxine abiotic siderophores for technetium complexation: radiolabeling and biodistribution studies

Julien Leenhardt^1,3^, Alexandre Biguet Petit Jean^2^, Florian Raes^1^, Emilien Nguessan^1^, Marlène Debiossat^1^, Clémence André^1^, Sandrine Bacot^1^, Mitra Ahmadi^1^, Nicolas de Leiris^1^, Loïc Djaileb^1^, Catherine Ghezzi^1^, Marie-Dominique Brunet^1^, Alexis Broisat^1^, Pascale Perret^1^, Amaury du Moulinet d'Hardemare^3^

1 Univ. Grenoble Alpes, INSERM, CHU Grenoble Alpes, LRB, 38000 Grenoble, France

2 CHU Saint-Etienne, Radiopharmacy / Nuclear medicine, Saint-Etienne, France

3 Univ. Grenoble Alpes, Department of Molecular Chemistry, Saint Martin d'Hères, France

Supplementary Information

Fig S1: [^99m^Tc]Tc(*O-*TRENOX)] chromatographic analysis on Whatman 0.16MM paper in a 4:1 Chloroform – Ethanol mixture


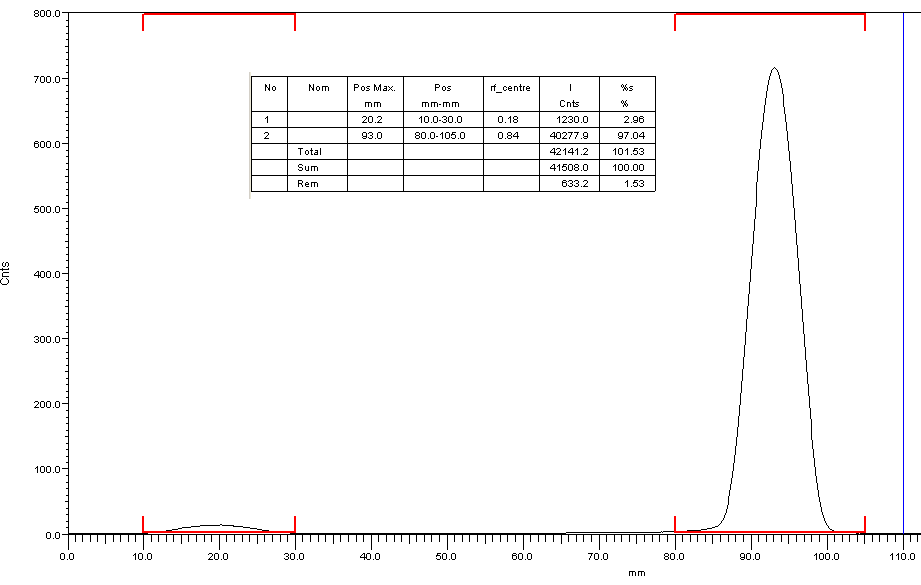


Fig S2: HPLC radiochromatogramm of [^99m^Tc]Tc(*O-*TRENOX)] (radioactive signal on the top and UV signal on the bottom)


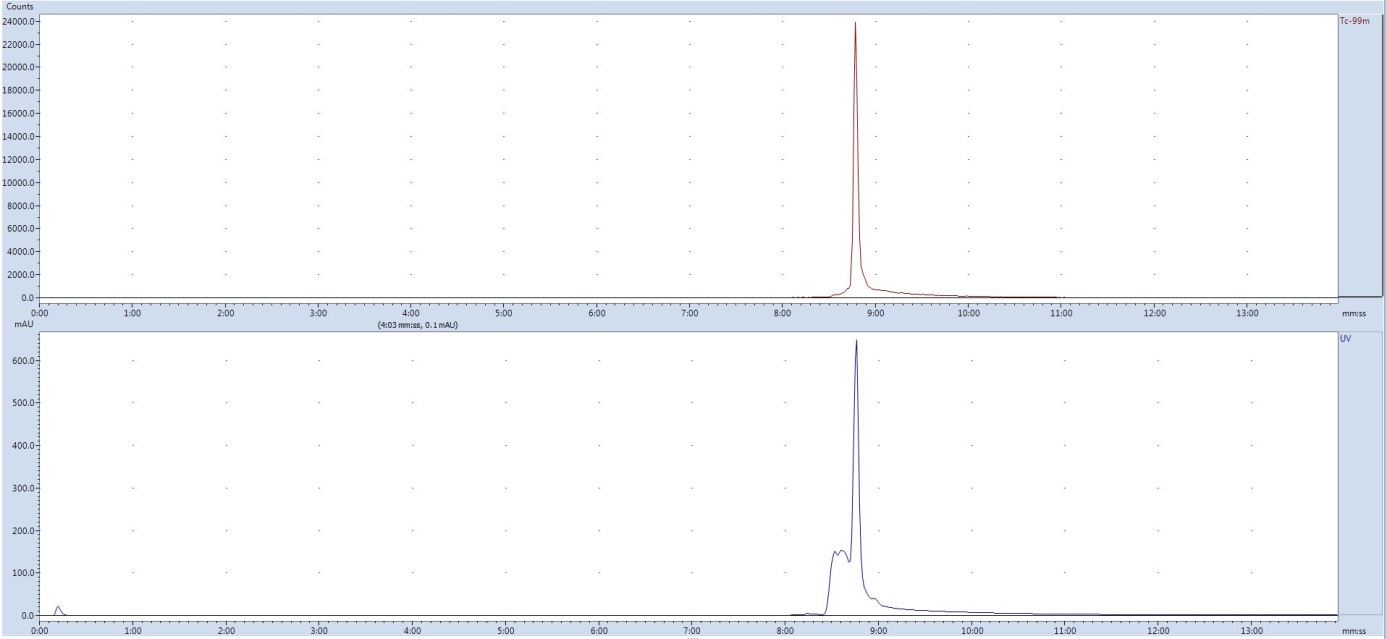


Fig S3: [^99m^Tc]Tc(*O-*TRENSOX)] chromatographic analysis on iTLC SG paper with sodium chloride 0.9% (top) and in a 3:2 ethyl-acetate – Methyl ethyl ketone (bottom)


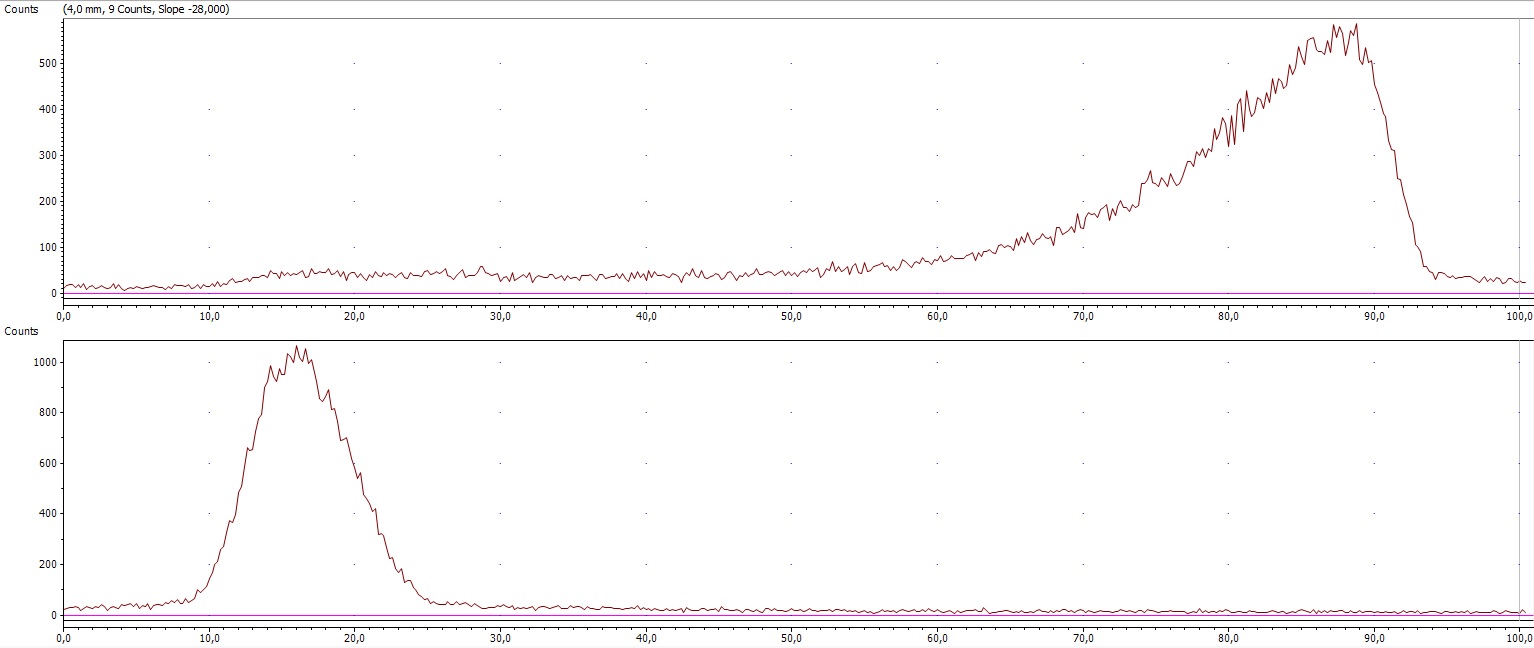


Fig S4: Comparative biodistribution of [^99m^Tc]Tc-*O-*TRENOX and [^99m^Tc]Tc-*O-*TRENSOX at each times (30, 60 and 240 minutes) (4 mice per chelate per times) formulated in %ID/g. *: p<0.05; ** p<0.01 and *** p<0.001 [^99m^Tc]Tc-*O-*TRENOX vs and [^99m^Tc]Tc-*O-*TRENSOX.


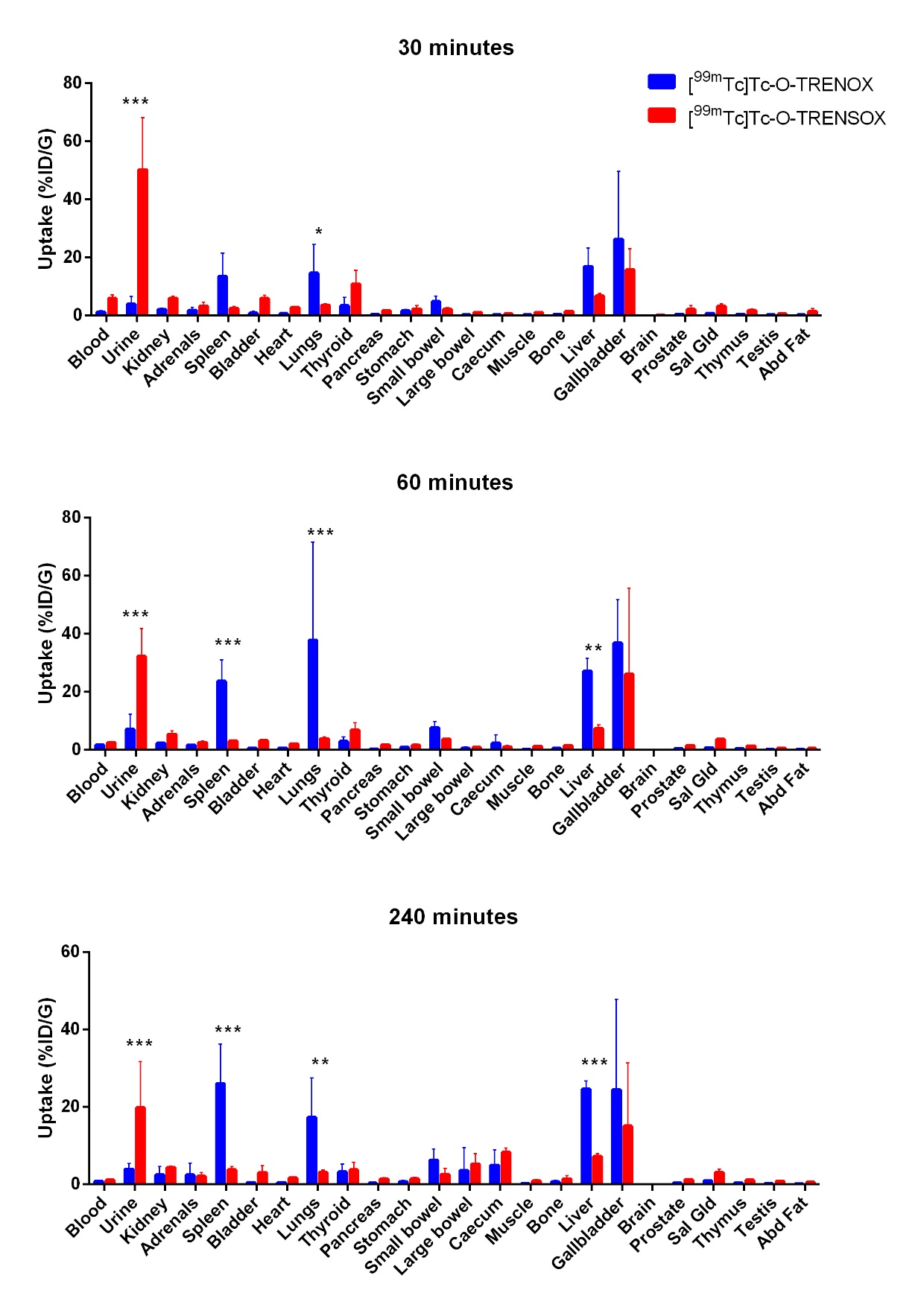


Fig S5 Representative SPECT-CT whole body imaging (sagittal, coronal and transversal views) with [99mTc]Tc-O-TRENOX at 240 min after injection (A) and [99mTc]Tc-O-TRENOX pulmonary uptake function of time from radiolabeling (B). White arrow: small spot in lungs, yellow arrow: liver, green arrow: intestine.


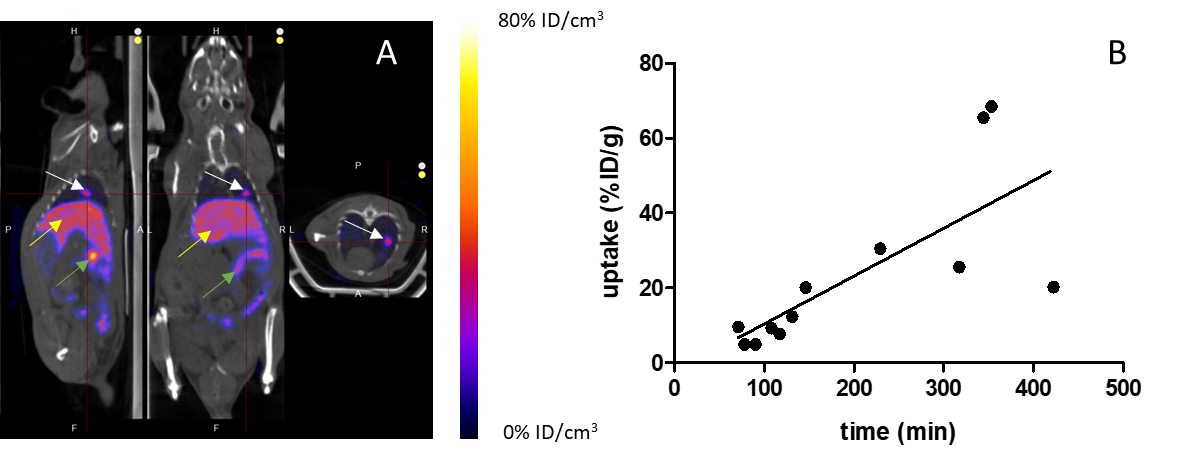

Supplement: Supplementary file 1 — Additional file 1. Fig. S1. [99mTc]Tc(O-TRENOX)] chromatographic analysis on Whatman 0.16MM paper in a 4:1 Chloroform – Ethanol mixture. Fig. S2. HPLC radiochromatogramm of [99mTc]Tc(O-TRENOX)] (radioactive signal on the top and UV signal on the bottom). Fig. S3. ([99mTc]Tc(O-TRENSOX)] chromatographic analysis on iTLC SG paper with sodium chloride 0.9% (top) and in a 3:2 ethyl-acetate – Methyl ethyl ketone (bottom). Fig. S4. Comparative biodistribution of [99mTc]Tc-O-TRENOX and [99mTc]Tc-O-TRENSOX at each times (30, 60 and 240 minutes) (4 mice per chelate per times) formulated in %ID/g. *: p<0.05; ** p<0.01 and *** p<0.001 [99mTc]Tc-O-TRENOX vs and [99mTc]Tc-O-TRENSOX. Fig. S5. Representative SPECT-CT whole body imaging (sagittal, coronal and transversal views) with [99mTc]Tc-O-TRENOX at 240 min after injection (A) and [99mTc]Tc-O-TRENOX pulmonary uptake function of time from radiolabeling (B). White arrow: small spot in lungs, yellow arrow: liver, green arrow: intestine. [file 41181_2023_214_MOESM1_ESM.docx]
